# Ultra-processed food consumption patterns among older adults in the Netherlands and the role of the food environment

**DOI:** 10.1007/s00394-020-02436-5

**Published:** 2020-11-24

**Authors:** Maria Gabriela M. Pinho, Jeroen Lakerveld, Marjolein C. Harbers, Ivonne Sluijs, Roel Vermeulen, Anke Huss, Jolanda M. A. Boer, W. M. Monique Verschuren, Johannes Brug, Joline W. J. Beulens, Joreintje D. Mackenbach

**Affiliations:** 1 Department of Epidemiology and Data Science, Amsterdam Public Health Research Institute, Amsterdam UMC, Vrije Universiteit Amsterdam, de Boelelaan 1089A, 1081 BT Amsterdam, The Netherlands; 2 Upstream Team, Amsterdam UMC, VU University Amsterdam, De Boelelaan 1089a, Amsterdam, The Netherlands; 3 Julius Center for Health Sciences and Primary Care, University Medical Center Utrecht, Utrecht University, Universiteitsweg 100, 3584 CG Utrecht, The Netherlands; 4 Faculty of Geosciences, Utrecht University, Princetonlaan 8a, 3584 CB Utrecht, The Netherlands; 5 Institute for Risk Assessment Sciences, Utrecht University, Yalelaan 1, 3584 CL Utrecht, The Netherlands; 6 National Institute for Public Health and the Environment, Antonie van Leeuwenhoeklaan 9, 3721 MA Bilthoven, The Netherlands; 7 Amsterdam School of Communication Research (ASCoR), University of Amsterdam, Nieuwe Achtergracht 166, 1018 WV Amsterdam, The Netherlands

**Keywords:** Dietary habits, Ultra-processed food, Community food environment, Obesogenic environment, Food retailers, Older adults

## Abstract

**Purpose:**

To describe the patterns of ultra-processed foods (UPFs) consumption in the Netherlands; to test if exposure to the food environment is associated with UPFs consumption; and if this association differed across educational levels and neighbourhood urbanisation.

**Methods:**

Cross-sectional study using 2015-data of 8104 older adults from the Dutch EPIC cohort. Proportion of UPFs consumption was calculated from a validated food-frequency questionnaire. Exposure to the food environment was defined as proximity and availability of supermarkets, fast-food restaurants, full-service restaurants, convenience stores, candy stores and cafés. Consumption of UPFs was expressed as both percentage of total grams and total kilocalories.

**Results:**

The study population was aged 70(± 10 SD) years and 80.5% was female. Average UPFs consumption was 17.8% of total food intake in grams and 37% of total energy intake. Those who consumed greater amounts of UPFs had a poorer overall diet quality. Adjusted linear regression models showed that closer proximity and larger availability to any type of food retailer was associated with lower UPFs consumption (both in grams and kilocalories). Somewhat stronger significant associations were found for proximity to restaurants (*β* = − 1.6%, 95% confidence interval (CI) = − 2.6; − 0.6), and supermarkets (*β* = − 2.2%, 95%CI = − 3.3; − 1.1); i.e., Individuals living within 500 m from the closest supermarket, as compared to 1500 m, had 2.6% less calories from UPFs. No differences were found on analyses stratified for urbanisation and education.

**Conclusions:**

Using various measures of exposure to the food environment, we found that exposure to restaurants and supermarkets was associated with somewhat lower consumption of UPFs.

**Electronic supplementary material:**

The online version of this article (10.1007/s00394-020-02436-5) contains supplementary material, which is available to authorized users.

## Background

An unhealthy diet is a leading risk factor for non-communicable disease and premature mortality [1]. Diets, especially in countries with a market-based economy, typically include a high percentage ultra-processed foods [2, 3]. Processed foods obtained from traditional methods of food processing such as fermentation to produce bread and cheese, the tinning of vegetables, and smoking of meats have been a part of people’s dietary habits for centuries, and contribute to the availability of safe, affordable and healthy diets. More recently, other methods of food processing have been introduced, like those referred to as ultra-processing. This includes industrial processes such as extrusion, pre-frying, and the addition of substances such as colour, stabilisers, artificial preservatives, flavours and flavour enhancers. Ultra-processing differs from traditional food processing in a number of ways, including its purpose, which is to create convenient, non-perishable food products that are ready-to-eat or heat, like frozen pizzas, chicken nuggets and instant sauces [4]. While *traditionally processed foods* are a part of a healthy diet, *ultra-processed foods* (UPFs) are generally energy dense, high in added sugar, fat and salt, and low in fibre, and, therefore, diminish diet quality [5–10]. Evidence from a randomized controlled trial suggests that diets including many ultra-processed foods lead to higher energy intake and higher body weight even after adjustment for sugar, fat, fibre, and other macronutrients content [11]. Recent research suggests that this increase in energy intake observed on diets rich in UPFs may be due to a faster energy intake rate (kcal/min) of UPFs [12]. Although causal mechanisms liking UPFs to health outcomes still need to be better understood, the consumption of UPFs has been associated with adverse health outcomes including obesity, metabolic syndrome, cancer, type II diabetes, cardiovascular diseases, and all-cause mortality [13–19].

Research based on national household budget surveys from nineteen European countries has shown that 26.4% of the total purchased dietary energy comes from ultra-processed foods. This percentage differs widely between countries, ranging from 10.2% in Portugal to 50.4% in the UK [16]. UPFs are generally heavily marketed and convenient, which contribute to their popularity and high intake levels [3, 20–22]. Another possible explanation for the high consumption of UPFs is its widespread availability in current food environments. It could well be that larger availability of UPFs pushes purchasing behaviour and results in higher consumption. The relation between the geographical availability and intake of UPFs has, however, not been explored in detail as of yet.

It is important to identify the individual and environmental factors that are associated with UPFs consumption to inform policymaking and design interventions to reduce the purchase and consumption of UPFs. Several studies that have focused on individual-level determinants have shown that higher consumption of UPFs was associated with male sex, younger age, lower education, and being a smoker [5, 6, 23]. A limited number of studies have also focused on environmental-level factors. Two Brazilian studies have shown that higher perceived availability of fruits and vegetables in the residential neighbourhood was associated with lower UPFs consumption [24]. Obtaining groceries in supermarkets rather than in local food shops has also been linked to higher UPFs purchasing [25]. In New Zealand supermarkets, UPFs were found to be the most prevalent type of packaged foods and showed a worse nutrient profile [7]. Although several studies provide evidence that the food environment is associated with dietary intake [26–28], research on the association between the objectively measured food environment and consumption of UPFs is lacking. Thus, in this study we want to address this gap by considering a broad range of food retailers that may be a source of UPFs purchases in the Netherlands, namely fast-food restaurants, full-service restaurants, supermarkets, and convenience stores.

Therefore, in this study we aim to (1) describe the pattern of UPFs consumption among older adults in the Netherlands and to explore how UPFs consumption relates to overall diet quality; (2) explore whether or not the availability of and proximity to different types of food retailers near the home is associated with consumption of UPFs. Since higher consumption of UPFs has been associated with lower educational attainment [6, 23]; and patterns of food consumption and health outcomes may differ for urban and rural areas [29, 30], we also aim to (3) explore if the associations we identify differ across levels of educational attainment and neighbourhood urbanisation.

## Methods

### Study design, sampling, and participants

In this cross-sectional study, we used data from the EPIC-NL cohort, a Dutch longitudinal cohort study on the role of lifestyle factors on chronic diseases. EPIC-NL encompasses the Prospect cohort and MORGEN cohort. The Prospect cohort consists of females aged 50–70 years at baseline from the region of Utrecht, recruited from the Dutch breast cancer screening program (*n* = 17,357). The MORGEN cohort consists of men and women aged 20–59 years at baseline from Amsterdam, Doetinchem, and Maastricht [31] (*n* = 22,654). Participants have been followed since baseline (1993–1997), at which point they had a physical examination and completed questionnaires. The study complies with the Declaration of Helsinki and was approved by the Institutional Review Board of the University Medical Center Utrecht and the Medical Ethical Committee of TNO Nutrition and Food Research. All participants provided informed consent [31].

In 2015, 14,949 participants were invited to complete a Food Frequency Questionnaire (FFQ), for which the response rate was 62.9%, resulting in 9399 participants with FFQ data. For the current analysis, participants were excluded if they had a ratio of energy intake to basal metabolic rate in the upper and lower 0.5% bounds (*n* = 93), if a valid residential address was not available, which was the case for all participants from Doetinchem (*n* = 1082), and if data for the participant was missing on educational attainment (*n* = 27), or marital status variables (*n* = 93). This resulted in an analytical sample of 8104 participants.

### Measures

#### Exposure to the food environment (independent variables)

The locations of food retailers were obtained from Locatus—a Dutch company that regularly collects information on the location of several types of retail outlets (https://locatus.com/en/). The location (*x* and *y* coordinates), type, size, and opening hours of all retailers were collected by Locatus staff via field audits. Yearly field audits are performed in shopping areas, while food retailers located outside shopping areas are audited every 2 or 3 years. In 2019, we tested the validity of Locatus data in terms of location and classification of food retailers against a field audit in selected areas across the Netherlands. We found an overall “good” to “excellent” agreement for both the location and classification of food retailers with a positive predictive value for location and classification of all food retailers was 0.90 [32].

Because the fourth EPIC-NL follow-up data collection was completed in 2015, we used Locatus data collected up to 2015. Given the focus of this study on the consumption of UPFs, we treated the following types of food retailers as exposure variables, as they are likely to be an important source of UPFs purchases in the Netherlands: fast-food restaurants, full-service restaurants, supermarkets, and convenience stores. Table 1 provides a description of food retailers comprising each of the analytical categories for food retailers.

**Table 1:** Categories of food retailers analysed

Analytical category	Food retailers composing the analytical category	Definition of food retailers and/or main food products offered by them
Fast-food restaurants	Chain or locally owned fast-food restaurant	Main provision of mostly deep-fried products that are ready for consumption in few minutes after ordering. Usually there is no table service available
	Food delivery and/or takeaway outlet	Main provision of meals that are not consumed in the store, but are collected or delivered
Full-service restaurants	Restaurant	Main provision of meals *a-la-carte*, table service is present. Drinks are only provided in combination with food
	Restaurant in hotel	Main provision of overnight accommodation in combination with an *a-la-carte* restaurant
	Café-restaurant	Main provision of both drinks and simple meals
Candy stores and cafés	Cake store	Main provision of cakes and pies
	Chocolate store	Almost exclusively provision of chocolate and pralines
	Ice-cream store	Almost exclusively provision of ice-cream
	Confectionery store	Main provision of candies and chocolates
Supermarket	Supermarket	Store selling a wide range of food and non-food products which are used on a daily basis. Store size should be at least 150 m^2^
Convenience stores	Convenience store	Same as supermarkets, but store size is less than 150 m^2^. This does not include stores in gas stations
Local food shops^a^	Greengrocer	Main provision of potatoes, vegetables and fruit
	Butcher	Main provision of meat and meat products
	Poultry shop	Main provision of poultry
	Bakery	Main provision of bread and pastries. Table service is possible, but this is not the main store activity
	Fish store	Main provision of fish, crustaceans and molluscs
	Delicatessen	Specialised store offering what are often higher-end food products
	Cheese store	Main provision of cheese

^a^Category used only as an adjustment for the broader food environment

**Fig. 1:**
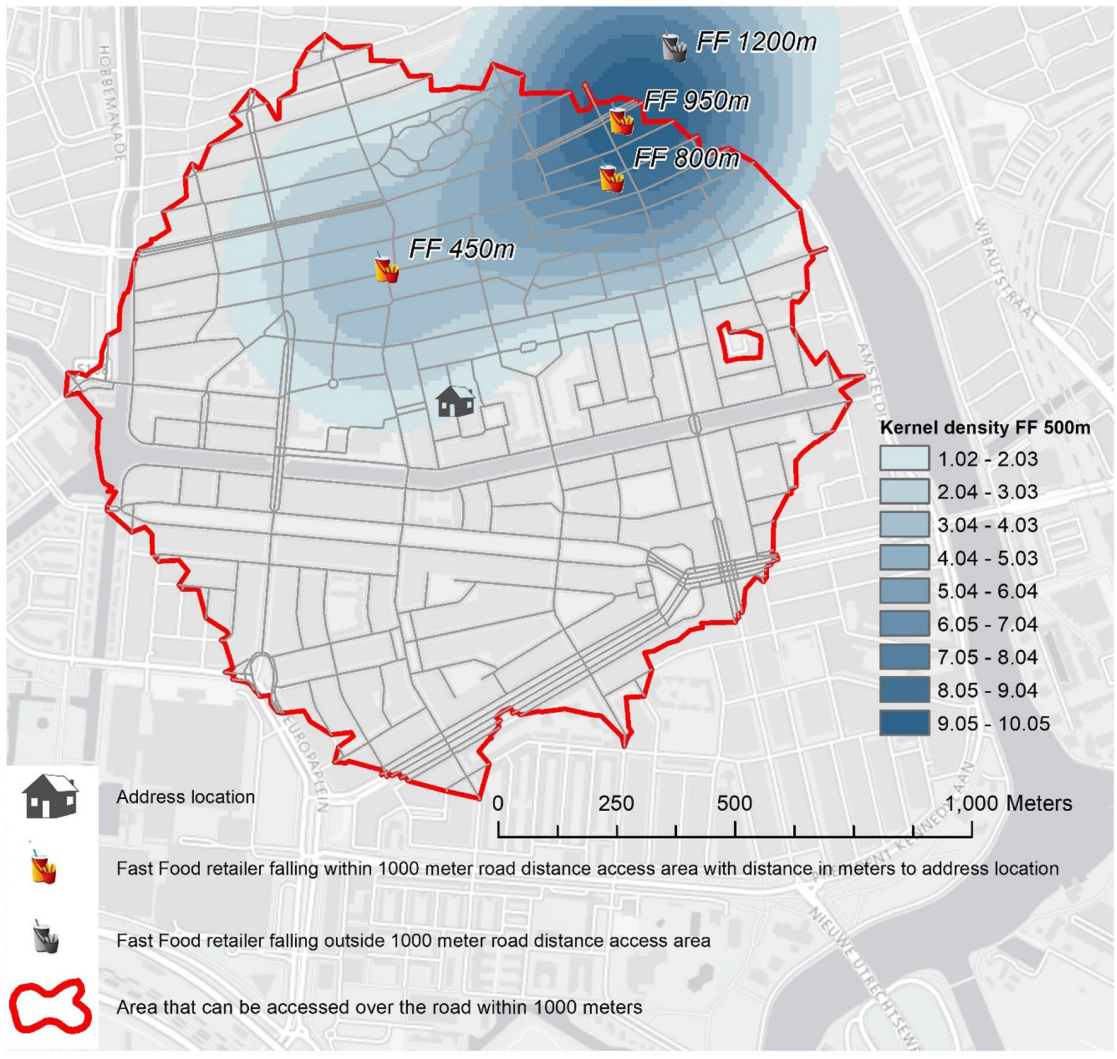
Representation of the three measures used to calculate exposure to the food environment. The figure exemplifies access to the fast-food restaurant within 1000 m street network buffer and 500 m kernels

Study results on the food environment-diet relationship tend to be inconsistent [33]. This may be due in part to uncertainty about which aspects of the food environment influence dietary choices and uncertainty about the food environments that individuals are exposed to during the whole day. To address these issues, we derived a range of food environment variables to account for these uncertainties. We used different buffer sizes that are commonly used and are likely to represent a reasonable area of exposure, and used accessibility (distance) and availability (density) measures to reflect different aspects of exposure to the food environment. In addition, more sophisticated operationalisation of exposures was used, such as street network analysis and kernel density estimates [34, 35]. Using ArcGIS version 10.4, with the geocoded addresses of participants as the reference point, we calculated: (1) an accessibility measure: the shortest distance along with a street network from the participants’ home address to the closest food retailer of each type, setting limit distances of 500, 1000, and 1500 m; these buffer sizes were chosen based on potential walking and cycling distances [36], and based on previous literature on food environment, diet and health behaviours [34, 37]; (2) an availability measure: counts of all food retailers of each type within a street-network calculated distance of 500, 1000, and 1500 m around the participants’ home address—analyses using street networks account for path barriers such as body of water, busy roads and train tracks; and (3) a weighted availability measure: kernel densities reflect weighted distances of all food retailers of each type around virtual grid cells of 100 square meter. The kernel density value for each cell is calculated from the centroid of the cell to its borders, therefore, density values are higher when more food retailers are clustered together [38]. The density value at a grid cell corresponding to an individual address was then attributed to that individual. Figure 1 shows a representation of the three measures used taking fast-food restaurants as an example. To understand method 1 and method 2 one should focus at the red street-network buffer of 1000 m around the home. According to the figure, the closest fast-food retailer is at 450 m from the individual home. Therefore, for method 1, that individual would receive a value of 450 m for the shortest distance along with a street network. If this individual walks 1000 m along with a street network from the home, he/she encounters 3 fast-food restaurants across this path. Therefore, for method 2, that individual would receive a count of 3. To understand method 3, one should focus on the 500 m blue kernel density buffer. We can see that the calculated kernel density value at the cell (100 square meter grid cells not shown in the picture) where the house is located ranges from 1.02 to 2.03. Therefore, for method 3, a value within this range is assigned to that individual. Since the variables were not normally distributed, we divided all variables into four categories. The street network, shortest distance variables were categorised as follows: 0–500 m; 500–1000 m; 1000–1500 m; and more than 1500 m. For the count variable and kernel density variables, the reference category was defined by individuals having no food retailer present in their home neighbourhood and tertiles of the remaining counts of retailers.

#### Dietary assessment

Participants completed a standardised, 160-item food frequency questionnaire (FFQ) developed for Dutch epidemiological studies, the FFQ-NL 1.0 [39]. This FFQ was previously validated against an average of 2.7 (range 1–5) telephone-based, 24-h recalls, as well as biomarkers from 24-h urine and blood samples. Results from the validation study showed Spearman rank correlation coefficients between the FFQ and 24-h recall estimates for the intake of 0.28 for potatoes; 0.53 for vegetables; 0.67 for fruits; 0.38 for meat; 0.28 for fish; 0.16 for cheese; 0.61 for dairy, excluding cheese; 0.38 for sweet products such as candies, chocolates and gums; and 0.33 for biscuits and pastry. Energy intake was estimated using the 2011 Dutch Food Composition table [40]. The dietary composition of food items in the FFQ-NL 1.0 was based on the food items as defined in the Dutch Food Composition database (NEVO), called ‘NEVO-codes’ [40]. The frequency of consumption of foods in the NEVO database by the Dutch population was determined in the Dutch National Food Consumption Survey [41]. To establish the FFQ items, the frequency of consumption of these foods were taken into account and expressed as a percentage. For instance, the FFQ food item “fried frozen potato, potato slices, croquettes, rösti”, is composed of three NEVO-codes and their respective frequency of consumption by the Dutch population: 77.8% of “unprepared potato slices/wedges”; 8.8% of “unprepared frozen potato croquettes”; and 13.3% of “unprepared frozen potatoes balls/wafers”.

For this study, all 1,283 NEVO-codes composing the FFQ-NL 1.0 were classified according to the NOVA classification [42]. Although the NOVA classification has been used in several studies, this is the first time that this classification was applied in this Dutch FFQ [39]. NOVA is a system for classification of foods according to the extent of their processing. NOVA proposes four food groups: “group 1—unprocessed or minimally processed foods”; “group 2—processed culinary ingredients”; “group 3—processed foods”; and “group 4—ultra-processed foods (UPFs)”. For the purposes of this analysis, we focused on “group 4—ultra-processed foods”. UPFs are defined as ‘industrial formulations made mostly from substances derived from foods (e.g., casein or hydrogenated oil), and additives such as flavour enhancers and food preservatives, with no, or very few intact ingredients from fresh foods [42]. This group includes products such as ready-to-eat or heat products including frozen pizza and lasagne, soft drinks, sweets, packaged snacks and chicken nuggets [42]. To classify the FFQ-NL 1.0 food items according to the NOVA classification, the frequency of consumption of each NEVO-code composing the FFQ food items was taken into account. For instance, as exemplified above, the FFQ food item “fried frozen potato, potato slices, croquettes, rösti”, is composed of three NEVO-codes and their respective frequency of consumption by the Dutch population: 77.8% of “unprepared potato slices/wedges”; 8.8% of “unprepared frozen potato croquettes”; and 13.3% of “unprepared frozen potatoes balls/wafers”. Since the first NEVO-code was classified as unprocessed or minimally processed foods and the two latter were classified as UPFs, the FFQ item “fried frozen potato, potato slices, croquettes, rösti”, was considered to be composed of 22.1% UPFs. When in doubt about the classification of a NEVO-code, we searched for this item (e.g., potato wafer) on the websites of Dutch supermarkets and checked the ingredient list of that food product. If the product contained ingredients such as wheat starch, and dextrose, emulsifier, this product would be classified as UPFs, according to the NOVA classification. After obtaining the percentage of UPFs in each FFQ food item, this value was used to calculate the percentage of grams per day and kilocalories per day of UPFs consumed by the participants. The percentage of grams in addition to the percentage of kilocalories was used to account for ‘diet’ and ‘light’ food and drink products with reduced caloric content.

To contextualize the consumption of UPFs in the Netherlands, and to explore how the consumption of UPFs relates to overall diet quality and consumption of individual food groups in the Netherlands, we also used the FFQ data to (1) obtain the frequency of consumption of selected food items that are presumably positively (i.e., processed meat, savoury snack, soft drinks) or negatively (i.e., fruit and vegetables) associated with consumption of UPFs; and (2) to calculate the participants’ score on the Dutch Healthy Diet index 2015 (DHD15-index) [43]. The index evaluates adherence to the Dutch dietary guidelines with scores in this study ranging from 0 (no adherence) to 130 (complete adherence).

#### Covariates

Energy intake was calculated from the FFQ data and it was added as a continuous covariate variable in the models. A self-administered participant questionnaire was used to collect individual socio-demographic characteristics that were used as covariates. Information on sex (male, female), and educational attainment were obtained at baseline. Information on age (continuous), region of residence (Amsterdam, Maastricht, or Utrecht), marital status, and energy intake (continuous) were obtained at follow-up. Marital status was categorised as ‘living with a partner’, or ‘not living with a partner (widow(er), divorced or single)’. Educational attainment was categorised as ‘lower educational attainment’ (primary to intermediate vocational education), ‘middle educational attainment (incomplete higher general secondary education to completed general secondary education)’, and ‘high educational attainment’ (higher vocational education to completed university). The Central Bureau of Statistics (CBS) in the Netherlands defines five categories for neighbourhood urbanisation defined as the number of addresses per km^2^. This was linked to the individuals based on the postcodes. Due to the variable distribution (few observations in the least urbanised category), we merged the two least urbanised categories and obtained a four-category urbanisation variable as follows: ‘very high urbanisation’, ‘high urbanisation, ‘moderate urbanisation’ and ‘low urbanisation’. Food retailers often co-locate, and previous research has suggested that analyses of the food environment should consider confounder adjustment for the broader food environment [44, 45]. Therefore, we adjusted our models for the presence of local food shops, which were less likely to sell UPFs, namely, green grocers, bakeries, delicatessens, cheese stores, nut stores, butchers, and fish stores.

#### Statistical analysis

Descriptive statistics are presented for sociodemographic characteristics and dietary intake for the total sample, and for the participants in the lowest and highest tertile of UPFs consumption as defined by grams and calories. We also present descriptive characteristics for the food environment variables, namely the distribution of variables as derived from closest network distance analysis (accessibility measure); counts of food retailers across a street network path and kernel density estimates at distances of 500, 1000, and 1500. Spearman’s rank correlation coefficients between the three exposure measures are presented in the supplementary files.

**Fig. 2:**
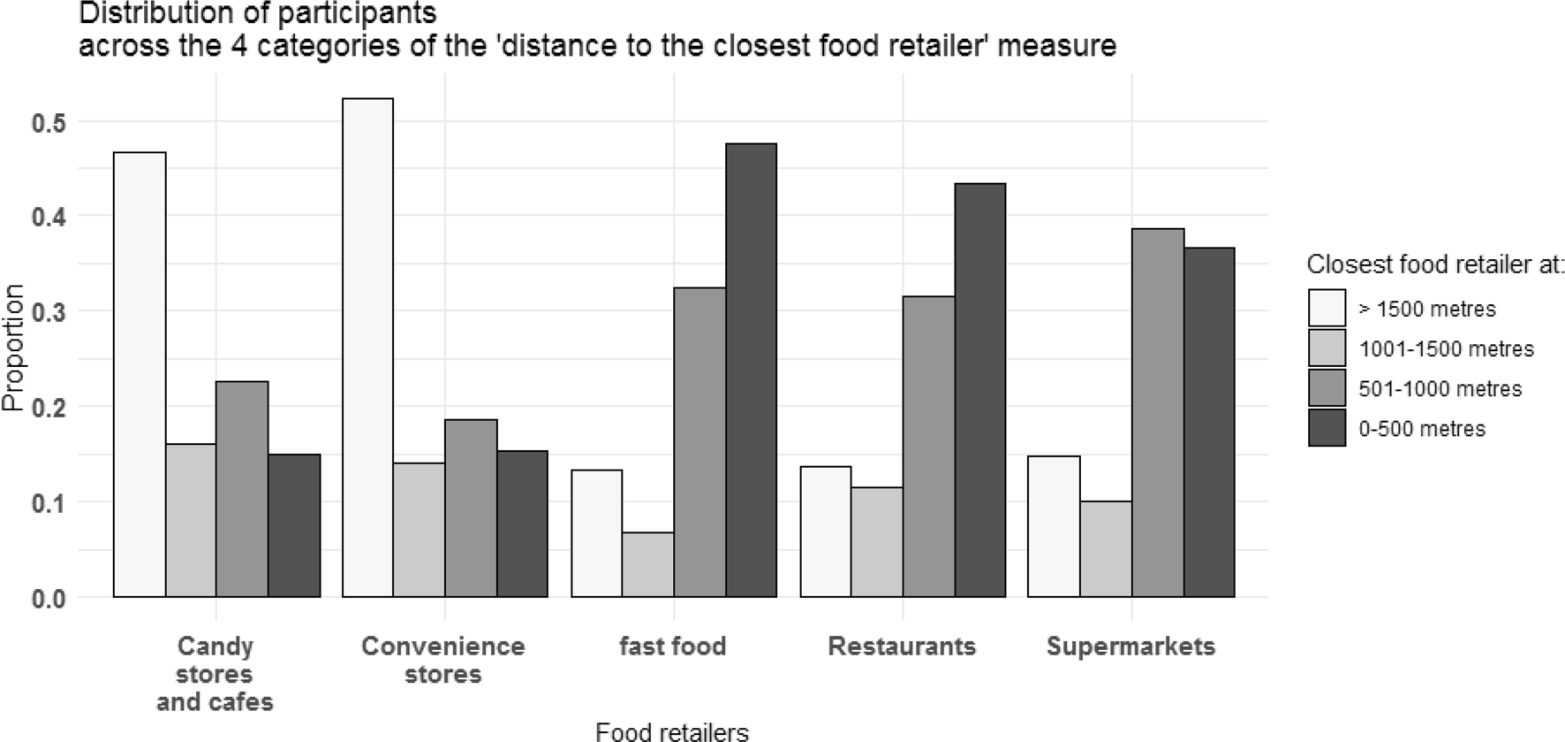
Descriptive characteristics for the food environment variables: distribution of participants across the categories of the ‘closest network distance’ measure

**Fig. 3:**
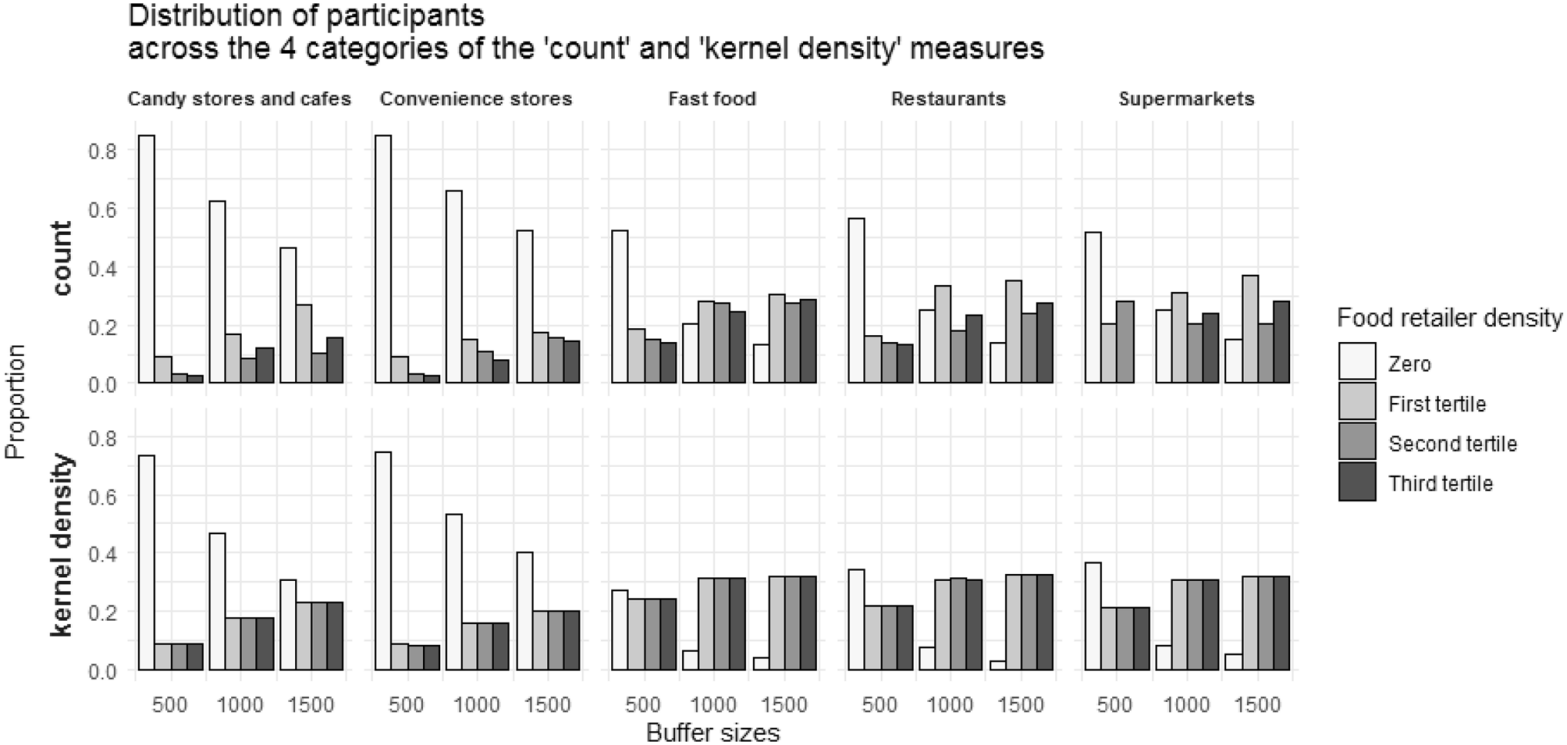
Descriptive characteristics for the food environment variables: distribution of participants across the categories of the ‘count’ and ‘kernel density’ measures

We used linear regression to test associations of objectively measured proximity to, counts of, and densities of each type of food retailer with the diet percentage of UPFs in grams and kilocalories adjusted for all covariates. For ease of presentation, we report the analysis using the 1000-m limit for the main analysis, and the 500 and 1500-m limits for sensitivity analyses in supplementary tables. Statistical significance was determined by the absence of zero in the 95% confidence interval. We tested effect modification for educational attainment and urbanisation levels by adding an interaction term between education and urbanisation and the food environment variables in separate models. To test whether or not the categories of the interaction term coefficients were jointly significant, we performed a post-estimation hypothesis test. We report a stratified analysis in the supplementary files when the overall interaction was significant (p-value lower than 0.05). Environmental variables and Fig. 1 were produced using ArcGIS® version 10.6.1. Statistical analyses were conducted in StataSE® version 14. Figures 2 and 3 were produced with the R package ggplot2 version 3.3.2.

## Results

Table 2 shows the descriptive characteristics of the participants. The mean age was 70 (± 10 Standard Deviation (SD)) years and the vast majority of participants were female (80.3%). Most participants were lower educated (57.4%) and lived with a partner (66.6%). The mean BMI was 25.8 (± 4.5 SD) kg/m^2^; and was lower among lowest consumers of UPFs: 25.0 kg/m^2^ (± 4.1 SD), as compared to the highest consumers: 26.7 kg/m^2^ (± 4.9 SD). Mean contribution of UPFs to total consumption was 18% (± 9% SD) in grams and 37% (± 11% SD) in calories. Median consumption of processed meat, savoury snacks, and soft drinks was higher among the highest consumers of UPFs, as compared to the lowest consumers. Median consumption of fruit and vegetables was lower among the highest consumers of UPFs. The mean score for the Dutch Healthy Diet index was 80 (± 17 SD), and those in the lowest tertile of consumption of UPFs, i.e., consuming less UPFs, scored higher (84.1; ± 17 SD) than those in the highest tertile of consumption of UPFs (73.7; ± 17 SD).

**Table 2:** Descriptive characteristics of the EPIC-NL participants (follow up wave 4)—total analytical sample and according to the level of intake of ultra-processed foods (UPFs)

	Total sample	Dietary percentage of grams from UPFs		Dietary percentage of calories from UPFs	
	Mean (SD) or % *n* = 8104	Lowest tertile *n* = 2702	Highest tertile *n* = 2701	Lowest tertile *n* = 2702	Highest tertile *n* = 2701
Age	69.9 (10.0)	70.7 (9.2)	68.8 (10.7)	70.4 (9.0)	69.5 (10.6)
Sex (%)					
Female	80.3%	87.4%	71.8%	83.6%	75.9%
Region of residence (%)					
Amsterdam	20.1%	21.8%	18.8%	22.9%	17.4%
Maastricht	23.6%	14.1%	35.0%	15.6%	32.1%
Utrecht	56.3%	64.1%	46.2%	61.5%	50.5%
Educational attainment (%)					
Lower	57.4%	48.5%	66.4%	48.0%	67.7%
Middle	11.0%	11.6%	10.2%	11.4%	10.2%
Higher	31.6%	40.0%	23.4%	40.6%	22.1%
Marital status (%)					
Living with partner	66.6%	64.2%	69.2%	63.3%	67.8%
BMI (kg/m^2^)^a^	25.8 (4.5)	25.0 (4.1)	26.7 (4.9)	25.1 (4.1)	26.6 (4.9)
Total calories (kcal)	1896 (640)	1679 (521)	2091 (721)	1731 (554)	2037 (695)
Percentage of calories from UPFs	37 (11)	28 (8)	46 (9)	25 (5)	49 (7)
Total grams	2535 (770)	2599 (746)	2508 (835)	2510 (760)	2521 (760)
Percentage of grams from UPFs	18 (9)	10 (2)	28 (9)	12 (5)	25 (10)
g/d of fruit^b^	163 (75–236)	211 (102–245)	115 (58–220)	208 (99–244)	115 (57–223)
g/d of vegetables^b^	119 (70–173)	135 (84–193)	102 (50–150)	135 (87–193)	100 (50–144)
g/d of processed meat^b^	23 (9–25)	16 (4–31)	32 (15–54)	17 (4–33)	31 (14–53)
g/d of savoury snacks^b^	7 (0–19)	3 (0–12)	12 (3–27)	3 (0–10)	13 (3–28)
g/d soft drinks^b^	0 (0–29)	0 (0–1)	27 (0–166)	0 (0–6)	15 (0–100)
Dutch Healthy Diet index 2015^c^	80 (17)	84 (17)	74 (17)	82 (17)	76 (16)

Percentages refer to participants in each category. Middle tertile for percentage of consumption of UPFs was omitted for the sake of space

Values are means (standard deviation), unless otherwise indicated

^**a**^Variable with missing data (*n* = 290)

^**b**^Median (interquartile range)

^**c**^Variable with missing data (*n* = 193)

*g/d* grams per day**, ***UPFs* ultra-processed foods, *BMI* body mass index

Figure 2 shows the distribution of participants across the categories of the accessibility measure (shortest network distance from home). Convenience stores and candy stores and cafes were less prevalent food retailers as only half of participants had these stores available within 1500 m from their home. Contrary, other food retailers were much more prevalent as 37% and 48% of participants had supermarkets and fast-food restaurants at less than 500 m from their home. Figure 3 shows the distribution of participants for the availability measures (counts of food retailers and kernel density), where a similar pattern was observed. For instance, considering the kernel density measure, while almost 80% of the participants had zero convenience stores within 500 m from their home, for fast-food restaurants this percentage was around 30% within 500 m, and as low as 4% within 1500 m. Knowledge about the fact that the distribution of participants across categories is different according to different food retailers may be relevant while choosing buffer sizes. As indicated in Figs. 2 and 3, to avoid large amounts of zeros in the first category, larger buffers are suggested for less prevalent food retailers such as convenience stores and cafes, while smaller buffers maybe used for more prevalent ones, such as restaurants, fast-food restaurants and supermarkets. The prevalence of food retailers in each study setting should be determined beforehand as different prevalence are likely to be found for different areas. More details regarding Figs. 2 and 3 can be found in Supplementary Table 1. Supplementary Table 2 shows the minimum and maximum count of food retailers in each tertile according to different distances in a network path from the participant’s home to each food retailer. Great variation was observed on the calculated street-network distances for different food retailer types. For instance, a minimum of 13 and maximum of 727 restaurants were encountered for a calculated 1500 m network path from home. In contrast, while travelling this distance a minimum of 5 and maximum of 30 supermarkets were encountered. Supplementary Table 3 shows the Spearman rank correlation coefficients for the three exposure measures. Correlations between Kernel density estimates and proximity to the closest food retailers had the lowest coefficients (bottom left of the matrix). In contrast, correlations between Kernel density estimates and counts within a network path had the highest coefficients (bottom middle of the matrix). Correlations of different measures of exposure to the same food retailer types were generally moderate to strong (*ρ* > 0.6).

Table 3 shows the results from the linear regression analysis using proximity to closest food retailers as exposure measure and the percentage of grams and calories consumed per day from UPFs as outcomes. In general, participants that lived closer to any food retailer, as compared to those that lived further away, consumed a lower percentage of grams and calories from UPFs. Regression coefficients were generally small, with strongest associations observed for restaurants (*β* = − 1.6%, 95%CI − 2.6; − 0.6), and supermarkets (*β* = − 2.2%, 95%CI − 3.3; − 1.1) when using the percentage of calories from UPFs as the outcome. Table 4 shows the results of the linear regression analyses using the counts of food retailers across a network distance of 1000 m, and kernel density estimates within a 1000 m radius, as exposure measures. Similar to the analysis using the proximity measure, living in areas that had *any* food retailers present was, in general, associated with a lower percentage of consumption in grams and calories from UPFs. More consistent trends with somewhat stronger coefficients were observed for counts of supermarkets and restaurants. Kernel density estimates, in turn, showed a slightly different pattern as associations were less often significant, effect sizes were smaller in some instances and the direction of the association for fast-food restaurants was positive, though no clear trend was observed across the categories.

**Table 3:** Regression coefficients (*β*) and 95% confidence intervals (95% CI) resulting from linear regression analyses with network distance to closest food retailers as exposure measure and the two outcomes: percentage of consumption in grams from ultra-processed food (UPFs) and percentage of consumption in kilocalories from UPFs (*n* = 8104)

Closest food retailers within a range of	Percentage of consumption from UPFs in	
	Grams	Kilocalories
	*β* (95% CI)	*β* (95% CI)
Fast-food restaurant		
> 1500 m	Ref.^a^	Ref.^a^
1001–1500 m	− 0.3 (− 1.4; 0.8)	− 1.4 (− 2.8; − 0.0)
501–1000 m	− 0.5 (− 1.4; 0.4)	− 0.6 (− 1.7; 0.5)
0–500 m	− 0.3 (− 1.2; 0.6)	− 0.8 (− 1.9; 0.4)
Convenience stores		
> 1500 m	Ref.	Ref.
1001–1500 m	0.2 (− 0.5; 0.8)	− 0.1 (− 0.8; 0.7)
501–1000 m	− 0.4 (− 0.9; 0.2)	− 0.8 (− 1.5; − 0.0)
0–500 m	− 0.4 (− 1.1; 0.2)	− 1.1 (− 2.0; − 0.3)
Restaurants		
> 1500 m	Ref.^a^	Ref.^a^
1001–1500 m	− 0.1 (− 1.0; 0.7)	− 0.5 (− 1.6; 0.6)
501–1000 m	− 0.8 (− 1.5; 0.0)	− 0.6 (− 1.5; 0.4)
0–500 m	− 1.6 (− 2.4; − 0.8)	− 1.6 (− 2.6; − 0.6)
Supermarket		
> 1500 m	Ref.	Ref.
1001–1500 m	− 1.2 (− 2.2; − 0.3)	− 1.1 (− 2.3; 0.1)
501–1000 m	− 1.8 (− 2.7; − 0.9)	− 1.8 (− 2.8; − 0.7)
0–500 m	− 2.1 (− 3.0; − 1.2)	− 2.2 (− 3.3; − 1.1)
Candy stores and cafés		
> 1500 m	Ref.^a^	Ref.^a^
1001–1500 m	0.1 (− 0.5; 0.7)	0.4 (− 0.3; 1.2)
501–1000 m	− 0.3 (− 0.8; 0.3)	− 0.3 (− 1.0; 0.3)
0–500 m	− 1.2 (− 1.9; − 0.6)	− 1.3 (− 2.1; − 0.4)

Coefficients were adjusted for age, sex, region of residency, educational attainment, urbanisation, marital status, total kilocalorie intake, and proximity to local food shops. Effect modification was tested in separate models by adding an interaction term between urbanisation and each of the proximity measure

^a^Indicates significant effect modification by urbanisation levels (*p* < 0.05)

**Table 4:** Regression coefficients (*β*) and 95% confidence intervals (95% CI) resulting from linear regression analyses with counts of food retailers within a network distance of 1000 m and kernel density estimates as exposure measure and the two outcomes: percentage of consumption in grams from ultra-processed food (UPFs) and percentage of consumption in kilocalories from UPFs (*n* = 8104)

		Counts within 1000-m street network		1000-m kernel density estimates	
		Percentage of consumption from UPFs in		Percentage of consumption from UPFs in	
		Grams	Kilocalories	Grams	Kilocalories
		*β* (95% CI)	*β* (95% CI)	*β* (95% CI)	*β* (95% CI)
Fast-food restaurant		^a^	^a, b^	^a^	^a^
Zero		Ref.	Ref.	Ref.	Ref.
First tertile		− 0.3 (− 0.9; 0.4)	− 0.2 (− 1.0; 0.6)	0.8 (− 0.2; 1.9)	0.9 (− 0.3; 2.2)
Second tertile		− 0.5 (− 1.3; 0.3)	0.1 (− 0.8; 1.1)	0.9 (− 0.2; 2.0)	1.9 (0.5; 3.2)
Third tertile		− 0.6 (− 1.6; 0.4)	− 0.7 (− 1.9; 0.5)	0.3 (− 0.9; 1.6)	0.9 (− 0.6; 2.5)
Convenience stores		^a^	^a^	^a^	^a^
Zero		Ref.	Ref.	Ref.	Ref.
First tertile		0.2 (− 0.4; 0.7)	− 0.2 (− 0.9; 0.5)	0.0 (− 0.6; 0.6)	0.1 (− 0.6; 0.8)
Second tertile		− 0.3 (− 1.0; 0.4)	− 0.6 (− 1.5; 0.3)	− 0.1 (− 0.7; 0.5)	− 0.3 (− 1.0; 0.4)
Third tertile		− 0.8 (− 1.7; 0.1)	− 1.7 (− 2.8; − 0.6)	− 0.3 (− 1.1; 0.4)	− 0.9 (− 1.8; − 0.1)
Restaurants				^a, b^	^a, b^
Zero		Ref.	Ref.	Ref.	Ref.
First tertile		− 0.8 (− 1.4; − 0.3)	− 0.4 (− 1.1; 0.3)	− 0.2 (− 1.0; 0.6)	0.5 (− 0.5; 1.5)
Second tertile		− 1.1 (− 18; − 0.3)	− 1.0 (− 1.9; − 0.1)	− 0.7 (− 1.6; 0.2)	− 0.0 (− 1.1; 1.1)
Third tertile		− 2.2 (− 3.0; − 1.3)	− 2.4 (− 3.4; − 1.4)	− 1.9 (− 2.9; − 1.0)	− 1.7 (− 3.0 − 0.5)
Supermarkets		^a,b^	^a,b^	^a^	^a^
Zero		Ref.	Ref.	Ref.	Ref.
First tertile		− 1.0 (− 1.6; − 0.4)	− 1.1 (− 1.9; − 0.3)	− 0.6 (− 1.6; 0.3)	− 0.1 (− 1.2; 1.1)
Second tertile		− 1.1 (− 1.8; − 0.4)	− 1.2 (− 2.1; − 0.3)	− 0.1 (− 1.2; 0.9)	0.4 (− 0.9; 1.6)
Third tertile		− 1.4 (− 2.2; − 0.5)	− 1.7 (− 2.7; − 0.7)	− 0.7 (− 1.8; 0.4)	− 0.3 (− 1.6; 1.1)
Candy stores and cafés			^a^	^a, b^	^a, b^
Zero		Ref.	Ref.	Ref.	Ref.
First tertile		− 0.2 (− 0.8; − 0.4)	− 0.2 (− 1.0; 0.5)	− 0.1 (− 0.7; 0.4)	0.3 (− 0.4; 1.0)
Second tertile		− 0.2 (− 1.0; 0.5)	− 0.1 (− 1.1; 0.8)	− 0.3 (− 0.9; 0.3)	0.1 (− 0.6; 0.9)
Third tertile		− 0.7 (− 1.5; 0.1)	− 1.7 (− 2.1; − 0.2)	− 0.5 (− 1.2; 0.2)	− 0.6 (− 1.5; 0.3)

Coefficients were adjusted for age, sex, region of residency, educational attainment, urbanisation, marital status, total kilocalorie intake, and proximity to local food shops. Effect modification was tested in separate models by adding an interaction term between education and each of the proximity measure; and between urbanisation and each of the proximity measure

^a^Significant effect modification by educational attainment (*p* < 0.05)

^b^Significant effect modification by urbanisation levels (*p* < 0.05)

A sensitivity analysis using the counts of food retailers and kernel density estimates within a distance of 500 and 1500 m showed a similar direction and strength of associations to the main analysis (Supplementary Table 4). Interaction terms with educational attainment were not significant for the analysis with proximity as exposure measure. However, a significant interaction was found with urbanization level in the models including proximity to restaurants and fast-food restaurants (Table 3). Significant interaction terms with educational attainment and urbanisation were also found in some analysis with counts and kernel density estimates (Table 4). However, analyses stratified for both urbanisation and education were mostly non-significant or similar to the general analysis (Supplementary Tables 5 to 9).

## Discussion

In this study, we describe patterns of UPFs consumption among a predominantly elderly and female Dutch population, and explored how the objectively measured residential food environment was associated with consumption of UPFs. Based on descriptive statistics, we found that participants that consume more UPFs were younger, more likely to be male, and lower educated. Furthermore, these individuals had a higher BMI, higher energy intake, consumed less fruits and vegetables, and more processed meats, savoury snacks and soft drinks. Closer proximity and larger availability to any type of food retailer was, in most instances, found to be associated with a lower consumption of UPFs, with somewhat stronger associations with exposure to restaurants and supermarkets. None of the food environment exposure variables were significantly associated with higher consumption of UPFs.

The Dutch dietary guidelines, whose adherence is measured by the Dutch Healthy Diet index, recommends the avoidance of foods such as processed meats and sugar-sweetened beverages. Therefore, we did expect that UPFs consumption and the Dutch Healthy Diet index would be inversely associated. However, it could also have been the case that those consuming UPFs such as sugar-sweetened beverages and ultra-processed meats, on the whole did have an adequate consumption of vegetables, whole grains and dairy thereby resulting in a relatively healthy overall diet quality. Nonetheless, descriptive statistics indicated that those who consumed diets containing a high percentage of UPFs had a lower score on the Dutch Healthy Diet index and consumed less fruit and vegetables, suggesting an overall lower diet quality. This finding is in line with previous research conducted in other countries [5, 6, 23], although what mechanism explains this association requires further investigation [12]. The proportion of UPFs in the diet in this population is comparable to that of populations from other European countries including France, Austria, and Norway [6, 16], but is lower than that of populations in the UK, Germany, and the USA [16, 23, 46]. However, it needs to be noted that the EPIC-NL cohort is, on average, an older and mostly female population. The dietary contribution of UPFs in younger adults, or in a more general adult population, is likely to be higher, as has been demonstrated by previous research [5, 6, 23].

We presumed that greater accessibility and availability of the food retailers included in the analysis might be a potential source of UPFs and would, therefore, be associated with higher UPFs consumption. However, the associations found were largely counter-intuitive, especially for food retailers such as candy stores and fast-food restaurants. Regarding supermarkets and restaurants, it could well be that our hypotheses were wrong and that the association for higher exposure to these food retailers and lower ultra-processed foods consumption would be the true association. Besides, the fact that the effect sizes found were generally small, could be an indication of a general null finding. However, a more likely explanation is that, despite the fact that we accounted for neighbourhood characteristics such as urbanisation and the presence of other food retailers, unmeasured/residual confounding could still have played a role [47, 48].

Inconsistent associations between the food environment and dietary intake have been reported previously [33] and have been attributed to factors such as the general use of low-quality instruments for dietary assessment, and oversimplification of the definition of exposure. That is an analysis that is restricted to the residential environment, which usually consider only simple measures that do not accurately reflect individuals’ exposure, and does not account for the broader food environment [33, 44, 49–51]. In this study, we attempted to avoid these potential pitfalls as much as possible. For instance, we used more comprehensive dietary data (i.e., data from a validated FFQ), employed different measures to define exposure, and used different distance categories. Because one cannot always determine in advance what measure would better capture different dimensions of exposure, we used several measures [23, 50]. Nonetheless, our results remained counterintuitive, thus, issues regarding our definitions of exposure do not seem to account for the unexpected findings of this study.

Correlation analysis showed that coefficients for different measures of exposure to the same food retailers were generally very strong, which could indicate that the various measures used represent exposure in the same way. However, when looking across different food retailers, we observed that kernel density estimates and counts within a network path correlated more strongly with each other than kernel density and distance to the closest food retailer. Indeed, Kernel density and counts within a street-network path are more complex measures than the closest distance measure, as the latter only takes into account proximity to one food retailer.

This study has both strengths and limitations. The fact that we only considered the residential environment may be a limitation [52]. However, the EPIC-NL cohort consists of a predominantly older female population, many of whom may be housewives or retired individuals. The residential setting is, therefore, more likely to be representative of exposure to their food environment than it would be for a younger population with greater mobility. As demonstrated by Kirkpatrick et al.’s systematic review, most studies that analyse the relationship between food environment and diet make use of short questionnaires for dietary assessment, which introduces a considerable source of bias in terms of assessing intake, thereby affecting the results of subsequent analyses [51]. In this context, the use of a comprehensive FFQ to obtain nutritional data is a strength of our study. However, any self-reported dietary data is still prone to bias (e.g., social desirability) and to both under and over reporting of dietary intake. The fact that we use different exposure measures of the food environment, accounting for both constructs of proximity and availability, and applied them to different types of food retailers, is also a strength. As has been suggested by previous reviews, more multi-method studies are needed to build a strong evidence base that identifies which measures apply to various contexts [35, 53]. Additional strengths of this study include the large sample size and the innovative aspects of the study, including being the first to report the consumption patterns of UPFs in the Netherlands, and the first to analyse the relationship with the objectively measured food environment.

In conclusion, in the present study among predominantly elderly and female participants, we found that those who consumed more UPFs had higher total energy intake and had a poorer overall diet quality. We did not find evidence that the accessibility or availability of five types of food retailers that offer many opportunities to purchase UPFs was associated with higher UPFs consumption in this population. On the contrary, using various measures of exposure to the food environment, we found evidence that exposure to some types of food retailers, especially restaurants and supermarkets, was consistently associated with somewhat lower consumption of UPFs.

## Electronic supplementary material

Below is the link to the electronic supplementary material.

Supplementary file 1 (DOCX 40 KB)

## Data Availability

Data on the location of food retailers are available from Locatus® but restrictions apply to the availability of these data, which were used under license for the current study, and so are not publicly available. Data are, however, available from the authors upon reasonable request and with permission of Locatus®. Internal rules apply to the use of EPIC-NL data, requests to work with the data are dependent on approval and should be sent to the cohort, for more information please visit https://www.epicnl.eu/.
