# Supplementary material for: Ultra-processed food consumption patterns among older adults in the Netherlands and the role of the food environment
Source: Eur J Nutr. 2020 Nov 24;60(5):2567–80. doi: 10.1007/s00394-020-02436-5 (PMC8275501; doi:10.1007/s00394-020-02436-5)
Supplement: Supplementary file 1 — (DOCX 40 KB) [file 394_2020_2436_MOESM1_ESM.docx]

**Supplementary Table 1.** Descriptive characteristics for the food environment variables: distribution of variables as derived from closest network distance analysis; counts of food retailers across a street network path and kernel density estimates at distances of 500, 1000, and 1500 metres (n=8,104).

| **Percentage of participants**  **across 4 categories of accessibility to the closest food retailer of each type ^a^** | |  | **Percentage of participants across 4 categories of counts of food retailers ^a^** | | | **Percentage of participants across 4 categories of density of food retailers (as derived from kernel density estimates) ^a^** | | |
| --- | --- | --- | --- | --- | --- | --- | --- | --- |
|  |  |  | **500m** | **1000 m** | **1500 m** | **500m** | **1000m** | **1500m** |
| **Fast-food restaurant** | **(%)** | **Fast-food restaurant (%)** | | **(%)** | **(%)** | **(%)** | **(%)** | **(%)** |
| > 1500 metres | 13.3 | Zero | 52.5 | 20.1 | 13.3 | 27.4 | 6.6 | 4.0 |
| 1001-1500 metres | 6.7 | First tertile | 18.6 | 28.0 | 30.6 | 24.4 | 31.1 | 31.9 |
| 501-1000 metres | 32.4 | Second tertile | 14.8 | 27.7 | 27.4 | 24.1 | 31.2 | 32.1 |
| 0-500 metres | 47.5 | Third tertile | 14.1 | 24.3 | 28.7 | 24.1 | 31.2 | 32.0 |
| **Convenience stores** |  | **Convenience stores** | |  |  |  |  |  |
| > 1500 metres | 52.2 | Zero | 84.8 | 66.2 | 52.2 | 74.8 | 53.1 | 40.1 |
| 1001-1500 metres | 14.0 | First tertile | 9.4 | 14.8 | 17.7 | 8.5 | 15.6 | 19.9 |
| 501-1000 metres | 18.6 | Second tertile | 3.4 | 10.7 | 15.8 | 8.4 | 15.7 | 20.0 |
| 0-500 metres | 15.3 | Third tertile | 2.4 | 8.2 | 14.3 | 8.4 | 15.7 | 20.0 |
| **Restaurants** |  | **Restaurants** | |  |  |  |  |  |
| > 1500 metres | 13.6 | Zero | 56.8 | 25.1 | 13.6 | 34.1 | 7.3 | 2.6 |
| 1001-1500 metres | 11.4 | First tertile | 16.4 | 33.2 | 35.0 | 22.0 | 30.7 | 32.3 |
| 501-1000 metres | 31.6 | Second tertile | 13.8 | 18.1 | 24.1 | 22.0 | 31.1 | 32.6 |
| 0-500 metres | 43.4 | Third tertile | 13.0 | 23.6 | 27.3 | 21.9 | 30.9 | 32.5 |
| **Supermarkets** |  | **Supermarkets** | |  |  |  |  |  |
| > 1500 metres | 14.8 | Zero | 51.6 | 24.9 | 14.8 | 36.9 | 8.2 | 5.1 |
| 1001-1500 metres | 10.0 | First tertile | 20.6 | 31.1 | 36.8 | 21.1 | 30.6 | 31.6 |
| 501-1000 metres | 38.6 | Second tertile | 27.8 | 20.1 | 20.6 | 21.0 | 30.6 | 31.7 |
| 0-500 metres | 36.6 | Third tertile | - ^b^ | 23.9 | 27.8 | 21.0 | 30.7 | 31.6 |
| **Candy stores and cafés** |  | **Candy stores and cafés** | |  |  |  |  |  |
| > 1500 metres | 46.6 | Zero | 85.2 | 62.7 | 46.6 | 73.2 | 46.6 | 30.6 |
| 1001-1500 metres | 16.0 | First tertile | 9.2 | 17.0 | 27.1 | 9.0 | 17.9 | 23.2 |
| 501-1000 metres | 22.5 | Second tertile | 3.0 | 8.5 | 10.5 | 9.0 | 17.8 | 23.1 |
| 0-500 metres | 14.9 | Third tertile | 2.5 | 11.9 | 15.8 | 8.9 | 17.7 | 23.1 |

Percentages refer to participants in each category. ^a^ First category represents the percentage of least-exposed individuals, either because they live further away from food retailers (accessibility measure), or because they there are no food retailers present at each of the distances. ^b^ Due to variable distribution, it was only possible to obtain three categories for this variable.

**Supplementary Table 2.** Minimum and maximum count of food retailers per category according to different calculated distances in a network path from the participant’s home to each food retailer

| Category | Fast-food restaurant | | | Convenience stores | | | Restaurant | | | Supermarket | | | Candy stores and cafés | | |
| --- | --- | --- | --- | --- | --- | --- | --- | --- | --- | --- | --- | --- | --- | --- | --- |
|  | Min - max | | | Min - max | | | Min - max | | | Min - max | | | Min - max | | |
|  | 500m | 1000m | 1500m | 500m | 1000m | 1500m | 500m | 1000m | 1500m | 500m | 1000m | 1500m | 500m | 1000m | 1500m |
| Zero | 0 | 0 | 0 | 0 | 0 | 0 | 0 | 0 | 0 | 0 | 0 | 0 | 0 | 0 | 0 |
| First tertile | 1 | 1-2 | 1-4 | 1 | 1 | 1 | 1 | 1-3 | 1-5 | 1-4 | 1 | 1-2 | 1 | 1 | 1-2 |
| Second tertile | 2-3 | 3-6 | 5-11 | 2 | 2-3 | 2-4 | 2-4 | 4-7 | 6-12 | 5-8 | 2 | 3-4 | 2 | 2 | 3 |
| Third tertile | 4-47 | 7-144 | 12-240 | 3-11 | 4-24 | 5-42 | 5-135 | 8-405 | 13-727 | - ^a^ | 3-18 | 5-30 | 3-11 | 3-33 | 4-50 |

^a^ Due to variable distribution, it was only possible to obtain three tertiles for this variable.

**Supplementary Table 3.** Spearman's correlation coefficient between the three exposure measures.

|  |  | (1) | (2) | (3) | (4) | (5) | (6) | (7) | (8) | (9) | (10) | (11) | (12) | (13) | (14) | (15) |
| --- | --- | --- | --- | --- | --- | --- | --- | --- | --- | --- | --- | --- | --- | --- | --- | --- |
| Closest fast food | (1) | 1.0 |  |  |  |  |  |  |  |  |  |  |  |  |  |  |
| Closest convenience store | (2) | 0.5 | 1.0 |  |  |  |  |  |  |  |  |  |  |  |  |  |
| Closest restaurant | (3) | 0.6 | 0.4 | 1.0 |  |  |  |  |  |  |  |  |  |  |  |  |
| Closest supermarket | (4) | 0.7 | 0.4 | 0.5 | 1.0 |  |  |  |  |  |  |  |  |  |  |  |
| Closest candy stores and cafés | (5) | 0.5 | 0.5 | 0.5 | 0.4 | 1.0 |  |  |  |  |  |  |  |  |  |  |
| Counts of fast food | (6) | 0.7 | 0.7 | 0.6 | 0.6 | 0.6 | 1.0 |  |  |  |  |  |  |  |  |  |
| Counts of convenience stores | (7) | 0.4 | 0.9 | 0.4 | 0.4 | 0.4 | 0.7 | 1.0 |  |  |  |  |  |  |  |  |
| Counts of restaurants | (8) | 0.5 | 0.5 | 0.8 | 0.5 | 0.6 | 0.7 | 0.6 | 1.0 |  |  |  |  |  |  |  |
| Counts of supermarkets | (9) | 0.6 | 0.5 | 0.6 | 0.7 | 0.6 | 0.7 | 0.6 | 0.7 | 1.0 |  |  |  |  |  |  |
| Counts of candy stores and cafés | (10) | 0.4 | 0.5 | 0.4 | 0.4 | 0.9 | 0.6 | 0.5 | 0.6 | 0.6 | 1.0 |  |  |  |  |  |
| KD fast food | (11) | 0.6 | 0.6 | 0.5 | 0.5 | 0.6 | 0.8 | 0.6 | 0.6 | 0.6 | 0.6 | 1.0 |  |  |  |  |
| KD convenience store | (12) | 0.4 | 0.9 | 0.4 | 0.3 | 0.4 | 0.6 | 0.9 | 0.5 | 0.5 | 0.4 | 0.6 | 1.0 |  |  |  |
| KD restaurants | (13) | 0.4 | 0.5 | 0.6 | 0.4 | 0.6 | 0.6 | 0.5 | 0.8 | 0.5 | 0.6 | 0.7 | 0.5 | 1.0 |  |  |
| KD supermarkets | (14) | 0.5 | 0.5 | 0.5 | 0.6 | 0.5 | 0.6 | 0.5 | 0.5 | 0.7 | 0.5 | 0.7 | 0.5 | 0.6 | 1.0 |  |
| KD candy stores and cafés | (15) | 0.4 | 0.4 | 0.4 | 0.3 | 0.8 | 0.6 | 0.4 | 0.6 | 0.5 | 0.8 | 0.6 | 0.5 | 0.6 | 0.6 | 1.0 |

KD = Kernel density; Each shade represents a different measure for the same food retailer type. For counts and kernel density estimates a buffer of 1000 metres was considered.

**Supplementary Table 4**. Regression coefficients (**β**) and 95% confidence intervals (95% CI) resulting from linear regression analyses with counts of food retailers within a network path and kernel density estimates, both in 500m and 1500m areas, as exposure measure and the two outcomes: percentage of consumption in grams from ultra-processed food (UPFs) and percentage of consumption in kilocalories from UPFs (n= 8,104).

|  | **Counts within a street network path** | | | | **Kernel density estimates** | | | | |
| --- | --- | --- | --- | --- | --- | --- | --- | --- | --- |
| **Percentage of consumption from UPFs in grams** | | | **Kilocalories** | | **grams** | | **Kilocalories** | | |
| **Fast-food restaurant** | **β (95% CI)** | **β (95% CI)** | **β (95% CI)** | **β (95% CI)** | **β (95% CI)** | **β (95% CI)** | **β (95% CI)** | **β (95% CI)** |  |
|  | **500m** | **1500m** | **500m** | **1500m** | **500m** | **1500m** | **500m** | **1500m** |  |
| Zero | REF | REF | REF | REF | REF | REF | REF | REF |  |
| First tertile | 0.3 (-0.2; 0.9) | -0.2 (-1.1; 0.6) | 0.2 (-0.5; 0.9) | -0.8 (-1.9; 0.2) | 0.0 (-0.6; 0.6) | 0.6 (-0.7; 1.8) | 0.5 (-0.2; 1.3) | 0.9 (-0.7; 2.4) |  |
| Second tertile | 0.1 (-0.6; 0.7) | -0.6 (-1.6; 0.4) | -0.1 (-0.9; 0.7) | -0.5 (-1.7; 0.7) | 0.2 (-0.5; 0.8) | 0.3 (-1.1; 1.7) | 1.1 (0.3; 1.9) | 1.5 (-0.2; 3.2) |  |
| Third tertile | -0.3 (-1.1; 0.5) | -0.7 (-1.9; 0.5) | -0.9 (-1.9; 0.1) | -1.2 (-2.7; 0.2) | -0.0 (-0.8; 0.8) | -0.1 (-1.6; 1.5) | 0.4 (-0.5; 1.4) | 0.5 (-1.4; 2.4) |  |
| **Convenience stores** | |  |  |  |  |  |  |  |  |
| Zero | REF | REF | REF | REF | REF | REF | REF | REF |  |
| First tertile | 0.1 (-0.6; 0.8) | 0.6 (0.1; 1.2) | -0.5 (-1.4; 0.4) | 0.1 (-0.6; 0.9) | 0.1 (-0.6; 0.8) | 0.1 (-0.5; 0.7) | -0.1 (-1.0; 0.7) | -0.3 (-1.0; 0.4) |  |
| Second tertile | -0.2 (-1.3; 0.9) | -0.1 (-0.8; 0.6) | -0.4 (-1.7; 1.0) | -0.1 (-1.0; 0.8) | 0.4 (-0.3; 1.1) | 0.3 (-0.3; 0.9) | -0.1 (-1.0; 0.8) | -0.1 (-0.8; 0.7) |  |
| Third tertile | -0.7 (-2.1; 0.6) | -0.3 (-1.1; 0.6) | -1.1 (-2.8; 0.5) | -1.7 (-2.8; -0.6) | -0.6 (-1.4; 0.2) | -0.0 (-0.8; 0.8) | -1.2 (-2.2; -0.3) | -0.9 (-1.9; 0.1) |  |
| **Restaurants** | |  |  |  |  |  |  |  |  |
| Zero | REF | REF | REF | REF | REF | REF | REF | REF |  |
| First tertile | -0.8 (-1.3; -0.2) | -0.6 (-1.4; 0.1) | -0.6 (-1.3; 0.1) | -0.7 (-1.6; 0.2) | -0.6 (-1.1; -0.0) | -0.1 (-1.4; 1.3) | -0.3 (-1.0; 0.4) | 0.3 (-1.3; 1.9) |  |
| Second tertile | -0.9 (-1.5; -0.2) | -1.3 (-2.2; -0.4) | -1.1 (-1.9; -0.3) | -1.1 (-2.2; 0.0) | -1.1 (-1.7; -0.5) | -0.5 (-1.9; 0.9) | -0.8 (-1.5; -0.1) | -0.1 (-1.8; 1.6) |  |
| Third tertile | -2.2 (-3.0; -1.3) | -1.4 (-2.5; -0.4) | -3.0 (-4.0; -2.0) | -1.5 (-2.8; -0.3) | -2.1 (-2.8; -1.4) | -1.3 (-2.8; 0.1) | -2.5 (-3.3; -1.7) | -1.4 (-3.2; 0.4) |  |
| **Supermarkets** | |  |  |  |  |  |  |  |  |
| Zero | REF | REF | REF | REF | REF | REF | REF | REF |  |
| First tertile | -0.7 (-1.3; -0.2) | -1.6 (-2.4; -0.8) | -0.2 (-0.8; 0.5) | -1.6 (-2.6; -0.6) | -0.2 (-0.7; 0.4) | -2.0 (-3.2; -0.7) | 0.2 (-0.6; 0.9) | -1.3 (-2.8; 0.3) |  |
| Second tertile | -1.3 (-1.9; -0.7) | -2.0 (-2.9; -1.0) | -1.4 (-2.2; -0.7) | -1.4 (-2.6; -0.2) | -0.3 (-0.9; 0.3) | -1.9 (-3.2; -0.5) | -0.3 (-1.0; 0.5) | -1.1 (-2.8; 0.5) |  |
| Third tertile | - ^a^ | -2.6 (-3.6; -1.5) | - ^a^ | -2.6 (-3.9; -1.3) | -0.1 (-0.8; 0.6) | -2.1 (-3.5; -0.6) | 0.1 (-0.7; 1.0) | -1.2 (-3.0; 0.6) |  |
| **Candy stores and cafés** | |  |  |  |  |  |  |  |  |
| Zero | REF | REF | REF | REF | REF | REF | REF | REF |  |
| First tertile | -0.7 (-1.4; -0.0) | 0.1 (-0.4; 0.6) | -0.6 (-1.4; 0.3) | 0.4 (-0.3; 1.0) | 0.1 (-0.6; 0.8) | 0.1 (-0.5; 0.7) | 0.3 (-0.5; 1.2) | 0.5 (-0.2; 1.2) |  |
| Second tertile | -1.4 (-2.5; -0.2) | -0.1 (-0.9; 0.7) | -1.2 (-2.7; 0.3) | -0.2 (-1.2; 0.7) | -0.6 (-1.3; 0.1) | -0.0 (-0.7; 0.6) | -0.3 (-1.2; 0.6) | 0.2 (-0.6; 1.0) |  |
| Third tertile | -1.0 (-2.4; 0.3) | -0.2 (-1.0; 0.6) | -1.6 (-3.3; 0.0) | -0.6 (-1.5; 0.4) | -1.0 (-1.8; -0.2) | 0.1 (-0.7; 0.8) | -1.3 (-2.3; -0.4) | 0.2 (-0.8; 1.1) |  |

Models were adjusted for age, sex, region of residency, educational attainment, urbanisation, marital status, total kilocalorie intake, and proximity to local food shops. ^a^ Due to variable distribution, it was only possible to obtain three tertiles for this variable.

**Supplementary Table 5**. Regression coefficients (**β**) and 95% confidence intervals (95% CI) resulting from linear regression analyses with counts of food retailers within a network path as exposure measure and the two outcomes: percentage of consumption in grams from ultra-processed food (UPFs) and percentage of consumption in kilocalories from UPFS, stratified by education attainment (n=8,104).

| **Counts within 1000 metres street network** | **Percentage of consumption from UPFs in grams** | | | **Percentage of consumption from UPFs in kilocalories** | | |
| --- | --- | --- | --- | --- | --- | --- |
|  | **Lowest education** | **Intermediate education** | **Highest education** | **Lowest education** | **Intermediate education** | **Highest education** |
|  | **n=** **4,648** | **n=** **892** | **n=** **2,564** | **n=** **4,648** | **n=** **892** | **n=** **2,564** |
|  | **β (95% CI)** | **β (95% CI)** | **β (95% CI)** | **β (95% CI)** | **β (95% CI)** | **β (95% CI)** |
| **Fast-food restaurant** |  |  |  |  |  |  |
| Zero | REF | REF | REF | REF | REF | REF |
| First tertile | -0.6 (-1.5; 0.4) | 0.7 (-1.2; 2.6) | 0.1 (-1.0; 1.2) | -0.9 (-2.0; 0.3) | 0.4 (-1.9; 2.7) | 0.8 (-0.6; 2.3) |
| Second tertile | -0.7 (-1.8; 0.4) | 0.2 (-2.0; 2.5) | -0.4 (-1.7; 0.9) | -0.8 (-2.1; 0.5) | 0.8 (-1.9; 3.6) | 1.8 (0.0; 3.5) |
| Third tertile | 0.0 (-1.3; 1.4) | -0.7 (-3.5; 2.0) | -1.2 (-2.7; 0.4) | -0.9 (-2.5; 0.8) | 0.4 (-3.0; 3.8) | -0.5 (-2.7; 1.6) |
| **Convenience stores** |  |  |  |  |  |  |
| Zero | REF | REF | REF | REF | REF | REF |
| First tertile | 0.4 (-0.4; 1.2) | 0.4 (-1.4; 2.3) | -0.3 (-1.2; 0.6) | 0.2 (-0.7; 1.2) | -1.1 (-3.4; 1.1) | -0.7 (-1.9; 0.6) |
| Second tertile | 0.1 (-0.9; 1.2) | -0.4 (-2.5; 1.7) | -0.9 (-2.0; 0.2) | 0.2 (-1.1; 1.4) | -0.5 (-3.0; 2.0) | -1.9 (-3.4; -0.5) |
| Third tertile | -0.4 (-1.8; 1.0) | -2.2 (-4.7; 0.2) | -0.5 (-1.8; 0.7) | -0.7 (-2.4; 0.9) | -1.2 (-4.2; 1.8) | -2.8 (-4.5; -1.0) |
| **Supermarket** |  |  |  |  |  |  |
| Zero | REF | REF | REF | REF | REF | REF |
| First tertile | -1.1 (-2.0; -0.3) | -0.1 (-1.8; 1.6) | -0.9 (-1.9; -0.1) | -1.4 (-2.4; -0.4) | 0.3 (-1.8; 3.2) | -0.8 (-2.1; 0.6) |
| Second tertile | -0.8 (-1.9; 0.2) | -0.6 (-2.7; 1.6) | -1.4 (-2.6; -0.3) | -1.3 (-2.5; -0.0) | -0.5 (-3.1; 2.2) | -1.2 (-2.8; 0.6) |
| Third tertile | -0.7 (-1.8; 0.5) | -2.1 (-4.4; 0.2) | -1.6 (-2.9; -0.3) | -1.2 (-2.6; 0.2) | -1.7 (-4.5; 1.0) | -2.1 (-3.9; -0.3) |
| **Candy stores and cafés** |  |  |  |  |  |  |
| Zero | - | - | - | REF | REF | REF |
| First tertile | - | - | - | -0.8 (-1.8; 0.2) | 1.4 (-0.6; 3.3) | 0.1 (-1.2; 1.2) |
| Second tertile | - | - | - | -0.1 (-1.4; 1.2) | 1.8 (-1.0; 4.5) | -0.7 (-2.3; 0.9) |
| Third tertile | - | - | - | -0.4 (-1.8; 1.0) | -0.7 (-3.4; 2.0) | -1.7 (-3.2; -0.2) |

Models were adjusted for age, sex, region of residency, marital status, urbanisation level, total kilocalorie intake, and proximity to local food shops. Empty cells indicate that no effect modification was found for that model.

| **1000-metre kernel density estimates** | **Percentage of consumption from UPFs in grams** | | | **Percentage of consumption from UPFs in kilocalories** | | |
| --- | --- | --- | --- | --- | --- | --- |
|  | **Lowest education**  **n=** **4,648** | **Intermediate education n=** **892** | **Highest education**  **n=** **2,564** | **Lowest education**  **n=** **4,648** | **Intermediate education**  **n=** **892** | **Highest education**  **n=** **2,564** |
|  | **β (95% CI)** | **β (95% CI)** | **β (95% CI)** | **β (95% CI)** | **β (95% CI)** | **β (95% CI)** |
| **Fast-food restaurant** |  |  |  |  |  |  |
| Zero | REF | REF | REF | REF | REF | REF |
| First tertile | -0.6 (-1.9; 0.7) | 0.9 (-1.7; 3.5) | 2.3 (0.9; 3.7) | -0.7 (-2.3; 0.8) | 0.5 (-2.6; 3.7) | 2.9 (1.0; 4.7) |
| Second tertile | -0.2 (-1.7; 1.2) | 1.1 (-1.8; 4.0) | 2.4 (0.8; 3.9) | 0.3 (-1.4; 2.0) | 1.4 (-2.1; 4.9) | 4.4 (2.3; 6.5) |
| Third tertile | 0.0 (-1.6; 1.6) | -0.2 (-3.5; 3.1) | 1.9 (0.1; 3.7) | 0.3 (-1.6; 2.2) | -0.1 (-4.1; 3.9) | 3.4 (1.0; 5.8) |
| **Convenience stores** |  |  |  |  |  |  |
| Zero | REF | REF | REF | REF | REF | REF |
| First tertile | 0.4 (-0.4; 1.2) | 0.7 (-1.0; 2.4) | -0.6 (-1.5; 0.3) | 0.8 (-0.2; 1.7) | -0.8 (-2.9; 1.2) | -0.6 (-1.9; 0.7) |
| Second tertile | 0.4 (-0.4; 1.3) | 0.9 (-0.8; 2.7) | -1.0 (-1.9; -0.1) | 0.3 (-0.7; 1.3) | -0.1 (-2.2; 2.0) | -1.0 (-2.2; 0.2) |
| Third tertile | 0.5 (-0.5; 1.6) | -0.7 (-2.7; 1.4) | -0.3 (-1.5; 0.8) | 0.1 (-1.1; 1.4) | -1.5 (-4.0; 1.0) | -1.0 (-2.5; 0.5) |
| **Restaurant** |  |  |  |  |  |  |
| Zero | REF | REF | REF | REF | REF | REF |
| First tertile | -1.1 (-2.2; -0.0) | 1.3 (-1.2; 3.7) | 1.1 (-0.2; 2.5) | -0.0 (-1.3; 1.3) | 0.0 (-3.0; 3.0) | 1.6 (0.0; 3.7) |
| Second tertile | -1.2 (-2.4; -0.1) | -0.6 (-3.1; 1.9) | 1.3 (-0.1; 2.7) | -0.4 (-1.8; 1.0) | -0.9 (-3.9; 2.2) | 2.1 (0.2; 4.1) |
| Third tertile | -1.8 (-3.1; -0.5) | -0.1 (-2.8; 2.7) | 0.0 (-1.5; 1.6) | -0.9 (-2.4; 0.7) | -1.2 (-4.6; 2.3) | -0.1 (-2.2; 1.9) |
| **Supermarket** |  |  |  |  |  |  |
| Zero | REF | REF | REF | REF | REF | REF |
| First tertile | -2.2 (-3.5; -1.0) | 1.4 (-0.9; 3.8) | 1.5 (0.2; 2.9) | -1.4 (-2.9; 0.1) | -0.2 (-3.0; 2.7) | 2.3 (0.5; 4.1) |
| Second tertile | -1.7 (-3.0; -0.4) | 2.4 (-0.2; 4.9) | 2.1 (0.7; 3.6) | -0.8 (-2.4; 0.7) | -0.1 (-3.2; 3.1) | 3.4 (1.5; 5.3) |
| Third tertile | -1.4 (-2.8; 0.0) | 0.4 (-2.4; 3.3) | 1.3 (-0.2; 2.9) | -0.3 (-2.0; 1.2) | -2.1 (-5.5; 1.4) | 1.7 (-0.4; 3.8) |
| **Candy stores and cafés** |  |  |  |  |  |  |
| Zero | REF | REF | REF | REF | REF | REF |
| First tertile | -0.3 (-1.1; 0.4) | 0.0 (-1.5; 1.6) | 0.5 (-0.4; 1.4) | 0.1 (-0.8; 1.1) | -0.6 (-2.5; 1.3) | 1.3 (0.1; 2.5) |
| Second tertile | -0.0 (-0.8; 0.8) | 0.5 (-1.2; 2.3) | -0.3 (-1.2; 0.6) | 0.3 (-0.6; 1.3) | 0.8 (-1.3; 2.9) | 0.5 (-0.7; 1.8) |
| Third tertile | 0.3 (-0.7; 1.2) | -0.5 (-2.5; 1.5) | -0.0 (-1.1; 1.0) | 0.3 (-0.8; 1.5) | -1.6 (-4.0; 0.9) | 0.1 (-1.3; 1.5) |

**Supplementary Table 6.** Regression coefficients (**β**) and 95% confidence intervals (95% CI) resulting from linear regression analyses with kernel density estimation as exposure measure and the two outcomes: percentage of consumption in grams from ultra-processed food (UPFs) and percentage of consumption in kilocalories from UPFs, stratified by education attainment (n=8,104).

Models were adjusted for age, sex, region of residency, marital status, urbanisation level, total kilocalorie intake, and proximity to local food shops.

**Supplementary Table 7**. Regression coefficients (**β**) and 95% confidence intervals (95% CI) resulting from linear regression analyses with network distance to closest food retailers as exposure measure and the two outcomes: percentage of grams from ultra-processed food (UPFs) and percentage of kilocalories from UPFs, stratified by urbanization levels (n=8,104).

| **Closest food retailers within a range of:** | **Percentage of consumption from UPFs in grams** | | | | **Percentage of consumption from UPFs in kilocalories** | | | |
| --- | --- | --- | --- | --- | --- | --- | --- | --- |
|  | **Very high urbanisation** | **High urbanisation** | **Moderate urbanisation** | **Low urbanisation** | **Very high urbanisation** | **High urbanisation** | **Moderate urbanisation** | **Low urbanisation** |
|  | **n=** **2392** | **n=** **2493** | **n=** **1481** | **n=** **1548** | **n=** **2392** | **n=** **2493** | **n=** **1481** | **n=** **1548** |
|  | **β (95% CI)** | **β (95% CI)** | **β (95% CI)** | **β (95% CI)** | **β (95% CI)** | **β (95% CI)** | **β (95% CI)** | **β (95% CI)** |
| **Fast-food restaurant** |  |  |  |  |  |  |  |  |
| > 1500 metres | REF | REF | REF | REF | REF | REF | REF | REF |
| 1001-1500 metres | 2.9 (-2.3; 8.0) | -1.2 (-3.7; 1.4) | 0.5(-2.0; 2.9) | -0.5 (-2.3; 1.2) | 1.2 (-5.4; 7.7) | -4.0 (-7.0; -0.9) | 1.0 (-2.1; 4.0) | -1.0 (-3.1; 1.1) |
| 501-1000 metres | -4.0 (-7.5; -0.5) | -1.9 (-4.2; 0.5) | 0.0 (-2.2; 2.1) | 0.8 (-0.6; 2.2) | -4.9 (-9.3; -0.4) | -2.2 (-5.1; 0.6) | 0.6 (-2.1; 3.2) | 0.2 (-1.5; 1.8) |
| 0-500 metres | -4.4 (-7.8; -1.0) | -1.1 (-3.5; 1.3) | 1.4 (-0.7; 3.5) | 0.0 (-1.4; 1.4) | -5.9 (-10.2; -1.5) | -1.8 (-4.7; 1.2) | 1.6 (-1.0; 4.2) | -0.6 (-2.3; 1.1) |
| **Restaurants** |  |  |  |  |  |  |  |  |
| > 1500 metres | REF | REF | REF | REF | REF | REF | REF | REF |
| 1001-1500 metres | -2.8 (-6.4; 0.8) | -2.4 (-4.2; -0.7) | 2.0 (0.2; 3.9) | 0.5 (-1.0; 2.1) | -3.8 (-8.3; 0.8) | -3.0 (-5.1; -0.8) | 2.2 (0.0; 4.5) | 0.0 (-1.9; 1.9) |
| 501-1000 metres | -4.0 (-7.3; -0.7) | -3.1 (-4.8; -1.4) | 1.3 (-0.3; 3.0) | -0.3 (-1.7; 1.0) | -4.3 (-8.4; -0.1) | -2.7 (-4.8; -0.6) | 1.7 (-0.3; 3.8) | -0.7 (-2.2; 0.9) |
| 0-500 metres | -5.8 (-9.1; -2.6) | -3.1 (-4.9; -1.3) | 0.9 (-0.7; 2.6) | -1.0 (-2.4; 0.3) | -6.4 (-10.5; -2.2) | -3.2 (-5.4; -1.0) | 1.4 (-0.7; 3.5) | -0.9 (-2.5; 0.7) |
| **Supermarket** |  |  |  |  |  |  |  |  |
| > 1500 metres | REF | REF | REF | REF | - | - | - | - |
| 1001-1500 metres | -3.2 (-7.3; 0.8) | -3.0 (-4.9; -1.1) | -0.2 (-2.3; 1.8) | 0.5 (-1.4; 2.4) | - | - | - | - |
| 501-1000 metres | -4.8 (-8.4; -1.3) | -3.6 (-5.4; -1.9) | 0.2 (-1.7; 2.1) | -0.3 (-1.7; 1.2) | - | - | - | - |
| 0-500 metres | -5.1 (-8.6; -1.5) | -4.0 (-5.8; 2.1) | 1.1 (-0.8; 3.0) | -2.1 (-3.7; -0.6) | - | - | - | - |
| **Candy stores and cafés** |  |  |  |  |  |  |  |  |
| > 1500 metres | REF | REF | REF | REF | REF | REF | REF | REF |
| 1001-1500 metres | 0.2 (-1.1; 1.5) | -0.5 (-1.5; 0.4) | 0.5 (-0.8; 1.8) | 0.3 (-1.6; 2.1) | 0.1 (-1.5; 1.7) | 0.3 (-0.9; 1.4) | 0.7 (-0.9; 2.2) | 0.6 (-1.6; 2.8) |
| 501-1000 metres | -0.4 (-1.6; 0.7) | -0.6 (-1.5; 0.4) | -0.5 (-1.8; 0.8) | 0.5 (-1.3; 2.3) | -0.3 (-1.8; 1.1) | -0.6 (-1.7; 0.5) | -0.9 (-2.4; 0.7) | -1.8 (-1.8; 2.5) |
| 0-500 metres | -1.6 (-2.8; -0.4) | -1.0 (-2.2; 0.2) | -2.1 (-3.8; -0.3) | 3.4 (0.7; 6.2) | -1.8 (-3.3; -0.3) | -0.5 (-2.0; 1.1) | -1.3 (-3.5; 0.9) | 0.6 (0.6; 7.1) |

Models were adjusted for age, sex, region of residency, educational attainment, marital status, total kilocalorie intake, and proximity to local food shops. Empty cells indicate that no effect modification was found for that model.

**Supplementary Table 8**. Regression coefficients (β) and 95% confidence intervals (95% CI) resulting from linear regression analyses with counts of food retailers within a network path as exposure measure and the two outcomes: percentage of consumption in grams from ultra-processed food (UPFs) and percentage of consumption in kilocalories from UPFs (n=8,104).

| **Counts within 1000 metres street network** | **Percentage of consumption from UPFs in grams** | | | | **Percentage of consumption from UPFs in kilocalories** | | | |
| --- | --- | --- | --- | --- | --- | --- | --- | --- |
|  | **Very high urbanisation** | **High urbanisation** | **Moderate urbanisation** | **Low urbanisation** | **Very high urbanisation** | **High urbanisation** | **Moderate urbanisation** | **Low urbanisation** |
|  | **n=** **2392** | **n=** **2493** | **n=** **1481** | **n=** **1548** | **n=** **2392** | **n=** **2493** | **n=** **1481** | **n=** **1548** |
|  | **β (95% CI)** | **β (95% CI)** | **β (95% CI)** | **β (95% CI)** | **β (95% CI)** | **β (95% CI)** | **β (95% CI)** | **β (95% CI)** |
| **Fast-food restaurant** |  |  |  |  |  |  |  |  |
| Zero | - | - | - | - | REF | REF | REF | REF |
| First tertile | - | - | - | - | -4.8 (-7.7; -1.8) | 0.6 (-0.9; 2.1) | 0.0 (-1.8; 1.8) | 0.3 (-1.1; 1.7) |
| Second tertile | - | - | - | - | -4.6 (-7.7; -1.5) | 0.8 (-0.9; 2.5) | 1.5 (-0.5; 3.4) | 0.1 (-2.0; 2.3) |
| Third tertile | - | - | - | - | -5.9 (-8.8; -2.4) | 0.7 (-1.4; 2.7) | -3.2 (-7.1; 0.6) | 0.5 (-15.7; 16.6) |
| **Supermarket** | - | - | - | - |  |  |  |  |
| Zero | - | - | - | - | REF | REF | REF | REF |
| First tertile | - | - | - | - | -1.4 (-3.9; 1.2) | -2.1 (-3.3; -0.9) | -1.1 (-0.4; 2.7) | -1.0 (-2.4; 0.5) |
| Second tertile | - | - | - | - | -2.4 (-5.0; 0.2) | -1.5 (-2.9; -0.1) | -0.5 (-1.5; 2.5) | -0.6 (-2.7; 1.5) |
| Third tertile | - | - | - | - | -3.2 (-5.8; -0.6) | -1.4 (-3.0; 0.2) | 1.4 (-1.2; 4.1) | -5.4 (-12.4; 1.7) |

Models were adjusted for age, sex, region of residency, educational attainment, marital status, total kilocalorie intake, and proximity to local food shops. Empty cells indicate that no effect modification was found for that model.

**Supplementary Table 9**. Regression coefficients (β) and 95% confidence intervals (95% CI) resulting from linear regression analyses with kernel density estimation as measure and the two outcomes: percentage of consumption in grams from ultra-processed food (UPFs) and percentage of consumption in kilocalories from UPFs, stratified by urbanization levels (n=8,104).

| **Kernel density estimates within 1000 metres street network** | **Percentage of consumption from UPFs in grams** | | | | **Percentage of consumption from UPFs in kilocalories** | | | |
| --- | --- | --- | --- | --- | --- | --- | --- | --- |
|  | **Very high urbanisation** | **High urbanisation** | **Moderate urbanisation** | **Low urbanisation** | **Very high urbanisation** | **High urbanisation** | **Moderate urbanisation** | **Low urbanisation** |
|  | **n=** **2392** | **n=** **2493** | **n=** **1481** | **n=** **1548** | **n=** **2392** | **n=** **2493** | **n=** **1481** | **n=** **1548** |
|  | **β (95% CI)** | **β (95% CI)** | **β (95% CI)** | **β (95% CI)** | **β (95% CI)** | **β (95% CI)** | **β (95% CI)** | **β (95% CI)** |
| **Restaurants** |  |  |  |  |  |  |  |  |
| Zero | REF | REF | REF | REF | REF | REF | REF | REF |
| First tertile | -5.8 (-16.7; 5.1) | -1.0 (-2.6; 0.5) | 0.2 (-1.7; 2.1) | 0.0 (-1.3; 1.2) | -6.6 (-20.4; 7.3) | 0.5 (-1.4; 2.4) | 1.5 (-0.8; 3.8) | -0.2 (-1.7; 1.4) |
| Second tertile | -8.0 (-19.0; 3.0) | -1.5 (-3.1; 0.2) | 0.0 (-1.9; 1.9) | -0.5 (-2.0; 0.9) | -8.2 (-22.1; 5.7) | -0.3 (-2.3; 1.7) | 1.4 (-1.0; 3.7) | -0.8 (-2.6; 0.9) |
| Third tertile | -9.2 (-20.2; 1.8) | -1.8 (-3.7; 0.0) | -1.9 (-4.2; 0.3) | -0.3 (-2.4; 1.9) | -10.4 (-24.3; 3.5) | -0.9 (-3.2; 1.4) | 0.0 (-2.7; 2.7) | -0.6 (-3.2; 2.0) |
| **Candy stores and cafés** |  |  |  |  |  |  |  |  |
| Zero | REF | REF | REF | REF | REF | REF | REF | REF |
| First tertile | 0.2 (-1.1; 1.4) | -0.4 (-1.2; 0.5) | -0.8 (-2.0; 0.4) | 0.5 (-1.2; 2.2) | 0.3 (-1.3; 1.8) | 0.0 (-1.0; 1.1) | 0.1 (-1.4; 1.6) | 1.1 (-0.9; 3.0) |
| Second tertile | -0.4 (-1.7; 0.8) | -0.7 (-1.7; 0.2) | -1.3 (-2.7; 0.0) | 1.7 (-0.1; 3.5) | 0.2 (-1.4; 1.7) | -0.4 (-1.6; 0.8) | -1.5 (-3.2; 0.2) | 2.1 (-0.1; 4.3) |
| Third tertile | -0.5 (-1.7; 0.8) | -0.6 (-1.9; 0.8) | -1.7 (-3.7; 0.4) | 1.4 (-1.4; 4.2) | -1.0 (-2.6; 0.6) | 0.1 (-1.6; 1.7) | -1.4 (-3.9; 1.1) | 1.4 (-1.9; 4.7) |

Models were adjusted for age, sex, region of residency, educational attainment, marital status, total kilocalorie intake, and proximity to local food shops. Empty cells indicate that no effect modification was found for that model.
